# Study of tau pathology in male rTg4510 mice fed with a curcumin derivative Shiga-Y5

**DOI:** 10.1371/journal.pone.0208440

**Published:** 2018-12-06

**Authors:** Daijiro Yanagisawa, Hamizah Shahirah Hamezah, Lina Wati Durani, Hiroyasu Taguchi, Ikuo Tooyama

**Affiliations:** Molecular Neuroscience Research Center, Shiga University of Medical Science, Otsu, Japan; Centre Hospitalier de l'Universite Laval, CANADA

## Abstract

Intracellular inclusions of aggregated tau appear in neurons and glial cells in a range of neurodegenerative diseases known as tauopathies. Inhibition of pathological changes in tau is a therapeutic target for tauopathy. We recently synthesized a novel curcumin derivative, named Shiga-Y5, and showed that Shiga-Y5 inhibited cognitive impairment and amyloid deposition in a mouse model of Alzheimer’s disease. Here we investigated whether Shiga-Y5 inhibited cognitive impairment and tau accumulation in a mouse model of tauopathy, rTg4510. The rTg4510 mouse is a bitransgenic mouse model that uses a system of responder and activator transgenes to express human four-repeat tau with the P301L mutation. This strain is obtained by crossing tetO-MAPT*P301L mouse line (on a FVB/NJ background) with CaMKII-tTA mouse line (on a C57BL/6J background). Male rTg4510 mice and wild-type mice were fed with a standard chow diet with or without Shiga-Y5 (500 ppm) for 4 months. Behavioral tests were conducted from 5.5 months of age, and the mice were sacrificed at 6 months of age. There were no significant changes in behavioral performance in rTg4510 mice fed with SY5-containing chow diet compared with rTg4510 mice fed with control chow diet. Histological and biochemical analyses also showed no significant alterations in tau accumulation by the treatment with SY5. One of noticeable finding in this study was that rTg4510 mice on a F1 female FVB/NJ x male C57BL/6J background showed more severe tau accumulation than rTg4510 mice on a F1 female C57BL/6J x male FVB/NJ background. Further studies to clarify the mechanisms underlying tau aggregation may help to develop therapeutic approaches aimed at preventing this pathological feature.

## Introduction

The microtubule-associated protein tau plays a role in stabilizing microtubules and promoting their self-assembly from tubulin subunits [1, 2]. Tau is normally a highly soluble protein and mainly found in axons in adult neurons, however, intracellular inclusions of abnormally modified aggregated tau appear in neurons and glial cells in a range of neurodegenerative diseases collectively known as tauopathies, which include Alzheimer’s disease (AD), progressive supranuclear palsy (PSP), corticobasal degeneration, Pick’s disease. The intracellular inclusions contain abnormally hyperphosphorylated tau self-assembled in a β-sheet conformation [1–3]. Mutations in the tau gene have been originally identified in patients with frontotemporal dementia and parkinsonism linked to chromosome 17 (FTDP-17) [4], and then many tau mutations were found in tauopathies, indicating that alterations of tau itself cause intracellular aggregation and neurodegeneration. It is therefore widely considered that inhibition of the pathological changes in tau is a therapeutic target in tauopathy.

Curcumin, a polyphenol, is a low molecular weight yellow-orange pigment derived from turmeric, that is from the rhizome of *Curcuma longa*. Curcumin has been reported to display binding to tau aggregates in human brain sections from patients with tauopathies such as AD and PSP [5]. Several studies indicated therapeutic effects of curcumin and its derivatives on tau clearance [6], tau aggregation [7, 8], and tau-induced neural injury [9] *in vivo*. We recently synthesized a novel curcumin derivative, 1,7-Bis(4’-hydroxy-3’-trifluoromethoxyphenyl)-4-methoxycarbonylethyl-1,6-heptadiene-3,5-dione, named as Shiga-Y5 (SY5), as a fluorine-19 magnetic resonance imaging probe to detect amyloid deposition[10]. In addition to 6 atoms of fluorine, SY5 bears a substitution at the C-4 position, which influences the ratio of keto to enol tautomers of curcumin and its derivatives and their effects on amyloid β (Aβ) aggregation[11]. When we investigated the effect of curcumin and SY5 on aggregation and cell toxicity of Aβ, both curcumin and FMeC1 modulated the formation of Aβ aggregates, however, only SY5 significantly attenuated cell toxicity of Aβ. In addition, treatment with SY5 improved cognitive impairment and inhibited amyloid deposition in an amyloid precursor protein with Swedish mutation (APPswe)/ the exon-9-deleted variant of presenilin 1 (PS1dE9) double transgenic mouse model of Alzheimer’s disease [11, 12]. Thus, it can be considered that SY5 may protect broad spectrum of protein aggregation and neuronal degeneration in vivo caused by other than Aβ, ex. tau protein. Here, the present study investigated whether SY5 had a therapeutic effect on cognitive impairments and tau accumulation in a bitransgenic mouse model of tauopathy, rTg4510, that reproduce both of tau aggregation and associated neuron loss[13]. While the results showed that SY5 had no pharmacological effects on the formation of tau pathology in rTg4510 mice, we found that the pathological phenotypes of rTg4510 mice were affected by the background strains those used for crossbred.

## Materials and methods

### Animals

This study was carried out in strict accordance with the recommendations in the Guide for the Care and Use of Laboratory Animals of the National Institutes of Health. The protocol was approved by the Committee on Animal Care of Shiga University of Medical Science (Number: 2017-7-11). All efforts were made to minimize suffering.

The rTg4510 mouse is a bitransgenic mouse model that uses a system of responder and activator transgenes to express human four-repeat tau with the P301L mutation in the forebrain with approximately 13-fold higher level than endogenous mouse tau [13, 14]. To obtain rTg4510 mice for this study, tetO-MAPT*P301L mice (on a FVB/NJ background; 015815, The Jackson Laboratory, Bar Harbor, ME, USA) carrying a responder transgene consisting of a tetracycline-operon–responsive element placed upstream of a cDNA encoding human four-repeat tau with the P301L mutation were crossed with mice from the CaMKII-tTA mouse line (on a C57BL/6J background; 007004, The Jackson Laboratory) carrying an activator transgene (tetracycline-controlled transactivator; tTA) that consisted of the tet-off open reading frame placed downstream of Ca2+-calmodulin kinase II promoter elements. We used the CaMKII-tTA mouse line on a C57BL/6J background, instead of the original 129S6 background [13, 14]. Consequently, rTg4510 mice on a F1 FVB/NJ x C57BL/6J background (female tetO-MAPT*P301L mouse line and male CaMKII-tTA mouse line) and on a F1 C57BL/6J x FVB/NJ background (female CaMKII-tTA mouse line and male tetO-MAPT*P301L mouse line) were used in the present study (Table 1). The introduction of the C57BL/6 strain into the rTg4510 mouse background has been reported to minimally alter the presentation of tau pathology of the original phenotype, without losing fidelity of the original phenotype [15]. Offspring were ear-punched and genotyped using polymerase chain reaction. Mice expressing neither the responder nor the activator were used as wild-type. Two to four mice were housed per standard laboratory cage on wood shavings. They were fed a standard chow diet and maintained at 23°C under a 12-h light/dark cycle (lights on for the hours 08:00–20:00) with free access to water and food in a specific pathogen free animal facility.

**Table 1:** Strain background of rTg4510 mice used in this study.

rTg4510	Background	Parents		Sex	Chow diet	
		Female	Male		Control	Shiga-Y5
rTg4510_TxC	F1 FVB/NJ x C57BL/6J	tetO-TauP301L (FVB/NJ)	CaMKII-tTA (C57BL/6J)	Male	4	5
rTg4510_CxT	F1 C57BL/6J x FVB/NJ	CaMKII-tTA (C57BL/6J)	tetO-TauP301L (FVB/NJ)	Male	4	2

### Study design

**Fig 1:**
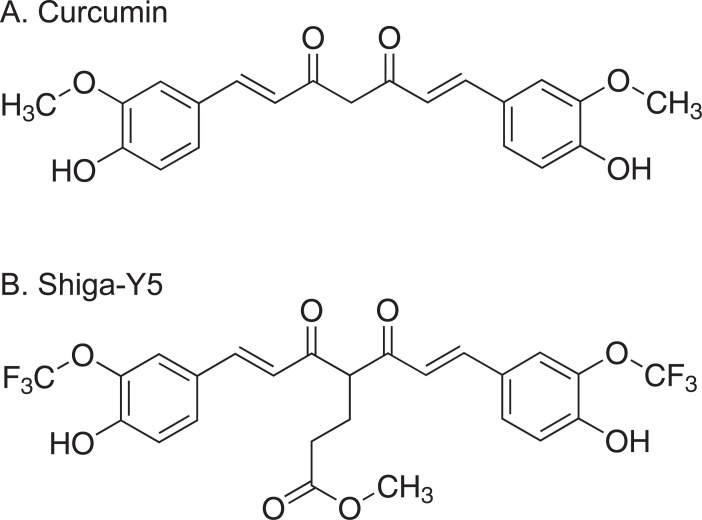
Chemical structures of curcumin and Shiga-Y5. Shiga-Y5 (B) is a curcumin (A) derivative bearing a methoxycarbonylethyl group and a trifluoromethoxy groups.

In this investigation 2-month-old male rTg4510 mice (n = 15) and 2-month-old male wild-type mice (n = 16) were used. Mice were fed with a standard chow diet (AIN-93M; Oriental Yeast, Tokyo, Japan) with or without SY5 (500 ppm which is equivalent to ~1.25 mg/day or 83 mg/kg body weight) (Fig 1) for 4 months. Behavioral tests were conducted from 5.5 months of age, and mice were sacrificed at 6 months of age as described below.

### Behavioral tests

#### Rotarod test

Motor activity and learning were assessed using a rotarod treadmill (MK-610A; Muromachi Kikai, Tokyo, Japan). Mice were placed on the rod with constant low-speed rotation. The time until the mouse fell from the rod was measured automatically. On day 1, mice were trained 5 times using the apparatus set to gradually increase rotation from 2 rpm to 20 rpm over 180 seconds. Performance on the rotarod test was measured 5 times per day using the apparatus set to accelerate from 2 rpm to 20 rpm over 180 seconds on day 2, and from 4 rpm to 40 rpm over 180 seconds on day 3.

#### Y-maze test

To assess spatial short-term memory performance and exploratory activity, a gray Y-maze test was used as described previously [12]. The maze consists of 21-cm long arms by 4-cm wide with 4-cm walls. Each animal received one trial, in the course of which the animal was placed into one of the three alleys and allowed free exploration of the maze for 8 min. The percent spontaneous alternation behavior was calculated as the ratio of entry into all three arms on consecutive choices in overlapping triplet sets. Higher percentages of spontaneous alternation behavior indicate better memory, while larger values of total arm entries indicate higher levels of activity.

#### Morris water maze test

The Morris water maze test was performed in a circular pool (120 cm in diameter, 30 cm high) as described previously [12]. The pool was filled with water (maintained at 20.0 ± 1.0°C), and was located in a large room with various distal visual cues. An invisible (transparent) circular platform (10 cm in diameter) was positioned 1.0 cm below the surface of the water and placed in the center of the south quadrant. The swimming activity of each mouse was monitored via a video camera mounted overhead, which relayed information including latency to find the platform, total distance traveled, and time and distance spent in each quadrant to a video tracking system (Muromachi Kikai, Tokyo, Japan).

Each mouse was given 5 trials per day at 20-min inter-trial intervals for 6 consecutive days to locate and climb on to the hidden platform. A trial was initiated by placing the mouse in the water with its nose directly facing the pool wall. The starting position varied between five constant locations at the pool rim. For each trial, the mouse was allowed to swim a maximum of 60 seconds to find the platform. When successful, the mouse was allowed a 20-seconds rest period on the platform. If unsuccessful within the allotted time period, the mouse was given a score of 60 seconds and then physically placed on the platform and also allowed the 20-secound rest period. On day 7, a single probe test was conducted for each study subject to measure spatial bias for previous platform location. This was accomplished by removing the platform from the pool and measuring the time spent in the previous platform quadrant location for 100 seconds.

Cued navigation test was performed using the platform shifted to another quadrant and raised 0.5 cm above the water surface on day 8. A red or white object was mounted on the platform extending approximately 7 cm above the water surface. To ensure that the mouse was using this proximal cue to locate the platform, the prominent visual features on the walls were removed and the starting points were pseudorandomly selected. Each mouse received 5 trials at 20-min inter-trial intervals. Again, the maximum searching time was 60 seconds and the mouse was allowed to remain on the platform for 20 seconds. Mice that did not find the platform within 60 seconds were gently guided to the platform. If the average latency of the last trial was more than 20 seconds, the mouse was excluded from the results of behavioral analyses because it was possible that the mouse had problems concerning the ability of eyesight, basic strategy (learning to climb on the platform) and the motivation (escape from water)[16].

### Preparation of brain extracts

Mice were sacrificed by overdose injection of sodium pentobarbital (200 mg/kg, i.p.). Brains were removed and cut into two hemispheres. Brain tissues of the cerebral cortex and the hippocampus were isolated from the right hemisphere, quickly frozen in liquid nitrogen, and stored at -80°C. Purification of sarkosyl-insoluble tau was performed as described previously [17] with slight modifications. Briefly, brain tissues (40 μg and 20 μg of the cerebral cortex and the hippocampus, respectively) were homogenized in 10 volumes of buffer H (10 mM Tris–HCl, pH 7.5 containing 0.8 M NaCl, 1 mM ethylene glycol tetraacetic acid, and 1 mM dithiothreitol) and centrifuged at 100,000 x g for 20 min at 4°C. The supernatant was collected as the TBS soluble fraction. The pellet was resuspended in buffer H and incubated in 1% Triton X-100 at 37°C for 30 min. Following the incubation, the sample were centrifuged at 100,000 x g for 20 min at 4°C. The pellet was resuspended in buffer H, incubated in 1% sarkosyl at 37°C for 30 min, and centrifuged at 100,000 x g for 30 min at 4 C. The supernatant was then collected (sarkosyl-soluble fraction). The detergent-insoluble pellet was extracted in 100 μL (cerebral cortex) and 50 μL (hippocampus) of urea buffer (8 M urea, 50 mM Tris-HCl, pH 7.5), sonicated, and centrifuged at 100,000 x g for 20 min at 4 C. The supernatant was then collected (sarkosyl-insoluble fraction).

### Western blotting

The protein concentration of extracts was determined by Protein Assay Bicinchoninate kit (Nacalai tesque, Kyoto, Japan). TBS-soluble and sarkosyl-insoluble fractions were run on precast polyacrylamide gels and transferred to polyvinylidene difluoride membranes. The membranes were blocked with 5% non-fat milk in Tris-buffered saline (25 mM Tris-HCl, pH 7.4, 0.9% NaCl) containing 0.1% Tween 20 (TBS-T) for 1 h at room temperature, incubated overnight with primary antibodies including mouse monoclonal antibodies against phosphorylated tau (Ser202 and Thr205) (clone AT8; 1:2000; Thermo Fisher Scientific, Waltham, MA, USA) and β-actin (1:5000; Santa Cruz Biotechnology, Dallas, TX, USA) and rabbit polyclonal anti-tau antibody (1:1000000; DAKO, Glostrup, Denmark) at 4°C, and further incubated with horseradish peroxidase-conjugated goat polyclonal antibody against mouse IgG (1:20000; Jackson ImmunoResearch, West Grove, PA, USA) or rabbit IgG (1:20000; Jackson ImmunoResearch) for 1 h. Immunoreactive proteins were visualized with chemiluminescence (SuperSignal West Pico; Thermo Fisher Scientific, Waltham, MA, USA) using a lumino-image analyzer (LAS-4000mini; Fujifilm, Tokyo, Japan). The band density was analyzed using an image processing software (Image J; National Institutes of Health, Bethesda, MD).

### Immunohistochemistry

The right hemisphere was fixed in 4% paraformaldehyde for 24 h at 4°C, immersed in 0.1 M phosphate buffer (pH 7.4) that contained 15% sucrose and 0.1% sodium azide for at least 2 days for cryoprotection, and subsequently cut into 20-μm sagittal sections in a cryostat. Free-floating sections were treated with 0.3% hydrogen peroxide in 0.1 M phosphate-buffered saline (PBS; pH 7.4) that contained 0.3% Triton X-100 (PBS-T) to eliminate endogenous peroxidase activity. After several washes, the sections were blocked with 2% bovine serum albumin (BSA) in PBS-T for 30 min at room temperature to block non-specific protein binding. The sections were then incubated with biotinylated antibodies against phosphorylated tau (clone AT8) (1:1000; Thermo Fisher Scientific) and tau (clone HT7) (1:2000; Thermo Fisher Scientific) in PBS-T containing 0.2% BSA overnight at 4°C, followed by avidin-biotin-peroxidase complex (Vectastain ABC Elite kit, 1:1000; Vector Laboratories, Burlingame, CA, USA) for 1 h at room temperature. All of the sections were washed several times with PBS-T between steps and labeling was accomplished through the use of 3,3’-diaminobenzidine (DAB; Dojindo Laboratories, Kumamoto, Japan), with nickel ammonium sulfate, which yielded a dark blue color. The sections were then mounted on glass slides and coverslipped with Entellan new mounting media (Merck Millipore, Billerica, MA). The sections were scanned using a camera, and the immunoreactive area was analyzed using ImageJ software for the quantitative analysis.

### Simplified Gallyas silver staining

A simplified Gallyas staining was performed according to methods used in a previous study [18]. Briefly, brain sections were mounted onto glass slides, briefly washed with water, and immersed in 0.3% potassium permanganate for 10 min. After washing with water, the sections were immersed in 2% oxalic acid for 2 min or until they turned white. The sections were further washed with water for another 6 minutes, immersed in alkaline silver iodide solution for 1 minute, washed three times with 0.5% acetic acid, and incubated in a freshly prepared developer solution for 18 minutes. After three washes with 0.5% acetic acid, the sections were further immersed in 0.5% hydrogen tetrachloroaurate (III) tetrahydrate for 5 minutes, lightly washed in water, immersed in 2% sodium thiosulfate for 1 minute, and finally washed again in water for a further 1 minute. Counter-staining was performed with hematoxylin. The sections were scanned using a camera, and the positively stained area was analyzed using ImageJ software for the quantitative analysis.

### Statistical analysis

Statistical analyses were performed in GraphPad Prism 7 (GraphPad Software, La Jolla, CA, USA). Data are presented as mean ± standard error of the mean (S.E.M.). Statistical significance was determined by Mann Whitney test for single comparisons, and two-way analysis of variance (ANOVA) followed by Tukey test for multiple comparisons. Statistical significance for training trials in the Morris water maze test was assessed with repeated-measures one-way ANOVA and Tukey test. P < 0.05 was considered statistically significant.

## Results

### Behavioral assessment of rTg4510 mice

In the cued navigation test performed on day 8 with Morris water maze test, two wild-type mice fed with control chow diet, one wild-type fed with SY5-containing chow diet and one rTg4510 mouse fed with control chow diet did not reach the platform within 20 seconds (S1 Fig). Since three of the wild-type mice reached the hidden platform within 20 seconds in the training trials, an indication of no problems concerning the ability of eyesight, basic strategy (learning to climb on the platform) and the motivation (escape from water), these mice were not excluded from the results of behavioral analyses. By these criteria, one rTg4510 mouse fed with control chow diet was excluded because of problems.

There were two factors (genotype x treatment) to affect the behavioral outcomes, and therefore we used two-way ANOVA followed by Tukey post test to see whether the outcomes were affected by treatment, by genotype, and/or by interaction. Results of statistical analysis from the behavioral tests in wild-type mice fed with control chow diet and SY5-containing chow diet and rTg4510 mice fed with control chow diet and SY5-containing chow diet are summarized in Table 2.

**Table 2:** Summary of statistical analysis in behavioral assessment.

Behavioral tests		Genotype		Treatment		Interaction	
		F (DFn, DFd)	P value	F (DFn, DFd)	P value	F (DFn, DFd)	P value
Rotarod	2–20 rpm	F (1, 26) = 0.387	P = 0.5393	F (1, 26) = 0.3468	P = 0.5610	F (1, 26) = 0.4191	P = 0.5231
	4–40 rpm	F (1, 26) = 1.399	P = 0.2476	F (1, 26) = 0.3207	P = 0.5761	F (1, 26) = 1.829	P = 0.1879
Y-maze	Total number of entries	F (1, 26) = 8.913	P = 0.0061	F (1, 26) = 2.336	P = 0.1385	F (1, 26) = 0.9959	P = 0.3275
	Spontaneous alteration	F (1, 26) = 0.03048	P = 0.8628	F (1, 26) = 0.3029	P = 0.5868	F (1, 26) = 0.04279	P = 0.8377
MWM	Probe test	F (1, 26) = 27.39	P<0.0001	F (1, 26) = 5.032	P = 0.0336	F (1, 26) = 0.3117	P = 0.5814

#### Motor activity and learning in rotarod test

**Fig 2:**
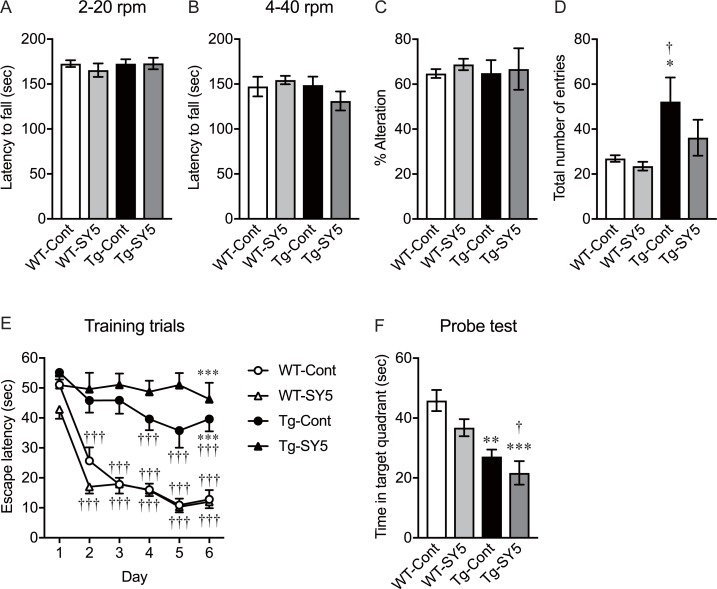
Behavioral assessment in rTg4510 mice. (A, B) Motor activity and learning were assessed using rotarod test with low (A) and high (B) speed settings. (C, D) Spontaneous alteration (C) and total number of entries (D) in Y-maze test was used to evaluate spatial short-term memory and exploratory activity, respectively. (E, F) Morris water maze test consisted of 5 training trials per day over 6 consecutive days (E) and probe test on day 7 (F). Wild-type mice fed with control chow diet (n = 8) and Shiga-Y5 (SY5)-containing chow diet (n = 8), and rTg4510 mice fed with control chow diet (n = 8) and SY5-containing chow diet (n = 7) were subjected to behavioral tests. According to the results of cued navigation test in Morris water maze test, one rTg4510 mouse fed with control chow diet was excluded from the results. Data are the mean ± standard of the mean. (A-F) Significance [Tukey multiple comparisons test after two-way ANOVA (genotyping x treatment)]: *p < 0.05, **p < 0.01, ***p < 0.001 vs. wild-type mice fed with control chow diet. (E) Significance (Tukey multiple comparisons test after repeated-measures one-way ANOVA): †††p < 0.001 vs. day 1.

Rotarod test was performed to assess motor activity and learning in wild-type mice fed with control chow diet and SY5-containing chow diet and rTg4510 mice fed with control chow diet and SY5-containing chow diet. There was no difference in the rotarod performance between the groups when the apparatus was set to accelerate from 2 rpm to 20 rpm over 180 seconds (Fig 2A). rTg4510 mice fed with control chow diet performed at the same level as wild-type mice fed with control chow diet when the speed and acceleration were increased to 4 to 40 rpm in 180 seconds (Fig 2B). Overall, there were no changes between the groups using this test.

#### Spatial short-term memory and exploratory activity in Y-maze test

Y-maze test was conducted to assess spatial short-term memory and exploratory activity in wild-type mice fed with control chow diet and SY5-containing chow diet, and rTg4510 mice fed with control chow diet and SY5-containing chow diet. The percentage of spontaneous alternation showed no difference between the groups (Fig 2C). Two-way ANOVA (genotype x treatment) indicated that there was a significant increase in total number of arm entries in rTg4510 mice, compared with wild-type mice [F (1, 26) = 8.913, p = 0.0061; Table 2). Post hoc analysis revealed that total number of arm entries was significantly increased in rTg4510 mice fed with control chow diet, compared with wild-type mice fed with control chow diet and SY5-containing chow diet (p < 0.05; Fig 2D). The increased number of arm entries was slightly but not significantly reduced in rTg4510 mice fed with SY5-containing chow diet (Fig 2D).

#### Spatial long-term memory and learning in Morris water maze test

In the Morris water maze test, latency to reach the platform in wild-type mice showed a significant reduction even at day 2, and there were significant differences in the latencies at day 3, 4, 5, and 6, compared with day 1 (p < 0.001; Fig 2E). rTg4510 mice fed with control chow diet showed a slight but significant reduction in the latencies to the platform at day 4, 5, and 6, compared with day 1 (p < 0.001), however, they took a significantly longer time to get to the platform at day 6, compared with wild-type mice fed with control chow diet (p < 0.001). rTg4510 mice fed with SY5-containing chow diet showed no reduction in the latencies to the platform during training trials and took significantly longer time to get to the platform at day 6, compared with wild-type mice fed with control chow diet (p < 0.001).

Two-way ANOVA (genotype x treatment) indicated that there was a significant reduction in time spent in the target quadrant in the probe test in rTg4510 mice, compared with wild-type mice [F (1, 26) = 27.39, p<0.0001; Table 2). Post hoc analysis revealed that the time spent in the target quadrant was significantly reduced in rTg4510 mice fed with control chow diet (p < 0.01) and SY5-containing chow diet (p < 0.001), compared with wild-type mice fed with control chow diet (Fig 2F). In addition, there was a significant difference between wild-type mice fed with SY5-containing chow diet and rTg4510 mice fed with SY5-containing chow diet (p < 0.05; Fig 2F). Two-way ANOVA (genotype x treatment) also indicated a significant difference in time spent in the target quadrant with and without the treatment [F (1, 26) = 5.032, p = 0.0336; Table 2), however, post hoc analysis revealed no significant difference between wild-type mice fed with control chow diet and SY5-containing chow diet, or rTg4510 mice fed with control chow diet and SY5-containing chow diet.

### Tau pathology in rTg4510 mice

**Fig 3:**
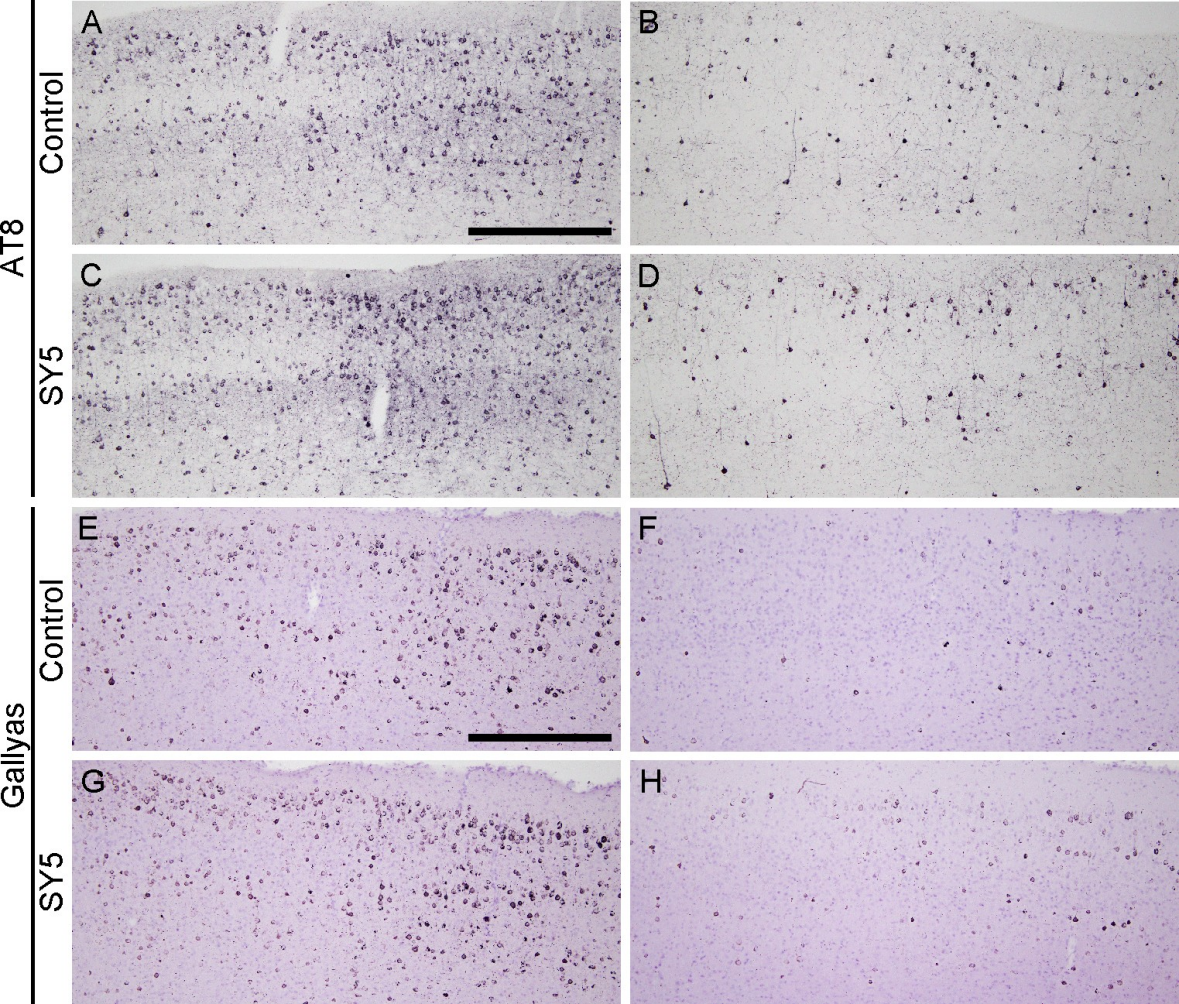
Tau pathology in the cerebral cortex in rTg4510 mice. Representative photographs showing the immunohistochemistry for AT8 (A-D) and Gallyas silver staining (E-H) in the cerebral cortex in rTg4510 mice fed with control chow diet (A, B, E, F) and Shiga-Y5 (SY5)-containing chow diet (C, D, G, H). Images of left and right columns were from rTg4510_TxC mice and rTg4510_CxT mice, respectively. Scale bars: 500 μm.

**Fig 4:**
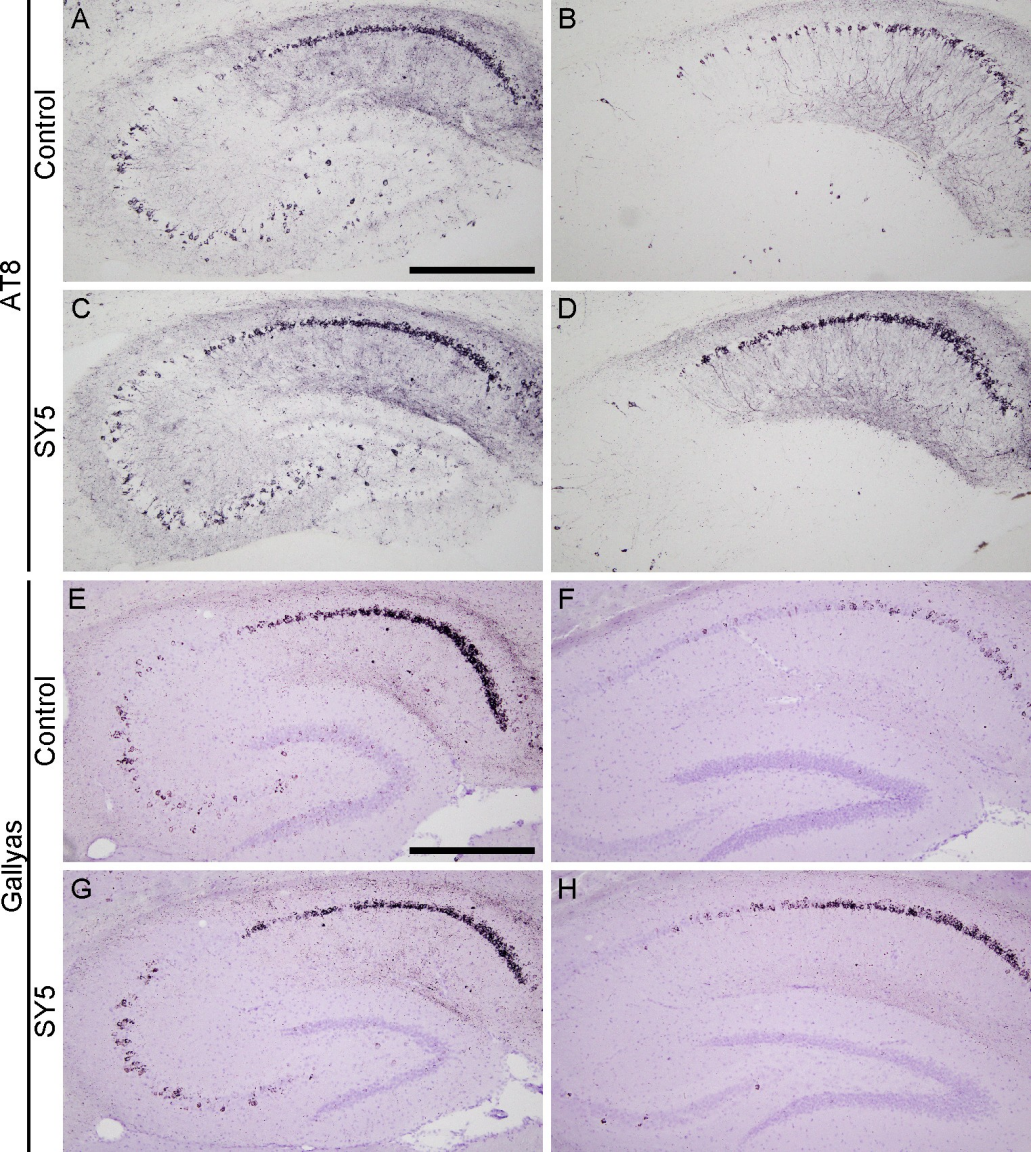
Tau pathology in the hippocampus in rTg4510 mice. Representative photographs showing the immunohistochemistry for AT8 (A-D) and Gallyas silver staining (E-H) in the hippocampus in rTg4510 mice fed with control chow diet (A, B, E, F) and Shiga-Y5 (SY5)-containing chow diet (C, D, G, H). Images of left and right columns were from rTg4510_TxC mice and rTg4510_CxT mice, respectively. Scale bars: 500 μm.

**Fig 5:**
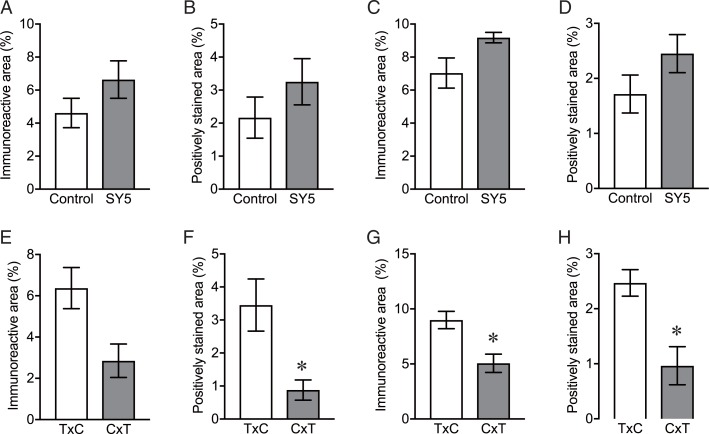
Quantification of the levels of tau pathology in rTg4510 mice. (A-D) AT8-mmunoreactive area (A, C) and Gallyas positively staining area (B, D) in the cerebral cortex (A, B) and hippocampus (C, D) in rTg4510 mice fed with control chow diet (n = 8) and Shiga-Y5 (SY5)-containing chow diet (n = 7) were analyzed. (E-H) The levels of AT8 (E, G) and Gallyas positive area in the cerebral cortex (A, B) and the hippocampus (C, D) were also analyzed in rTg4510_TxC mice fed with control chow diet (n = 4) and rTg4510_CxT mice fed with control chow diet (n = 4). Data are the mean ± S.E.M. Significance (Mann Whitney test): *p < 0.05 vs. rTg4510_TxC mice.

Brain sections in rTg4510 mice were examined by immunohistochemistry using AT8, and by Gallyas silver staining. AT8-immunoreactivity and argentophilic structures were detected in the forebrain region including the cerebral cortex (Fig 3) and the hippocampus (Fig 4). No statistical differences were found in the immunoreactivity and the argentophilic structures between rTg4510 mice fed with control chow diet and SY5-containing chow diet although slight increases were observed in rTg4510 mice fed with SY5-containing chow diet (Fig 5A–5D).

Interestingly, we identified two subgroups of animals with different degrees of severity of tau pathology (Figs 3 and 4). The group of mice with more intense tau pathology consisted of rTg4510 mice on a F1 FVB/NJ x C57BL/6J background (produced by crossing female tetO-MAPT*P301L mouse line with male CaMKII-tTA mouse line), referred to as rTg4510_TxC (Table 1 and left panels in Figs 3 and 4). The group of mice with less intense tau pathology consisted of rTg4510 mice on a F1 C57BL/6J x FVB/NJ background (produced by crossing female CaMKII-tTA mouse line and with tetO-MAPT*P301L mouse line), referred to as rTg4510_CxT (Table 1 and right panels in Figs 3 and 4). Quantitative analyses showed that the levels of AT8-immunoreactivity and Gallyas-positive structure in the cerebral cortex and striatum in rTg4510_TxC mice were twice or more of that of rTg4510_CxT (Fig 5E–5H).

### Soluble and insoluble tau in rTg4510 mice

Tau levels in TBS-soluble fractions and sarkosyl-insoluble fractions were measured by western blotting with statistical analysis between rTg4510 mice fed with control chow diet and SY5-containing chow diet. In addition, statistical analyses of tau levels between rTg4510_TxC mice fed with control chow diet and rTg4510_CxT mice fed with control chow diet were performed.

#### TBS-soluble tau levels in rTg4510 mice fed with Shiga-Y5

**Fig 6:**
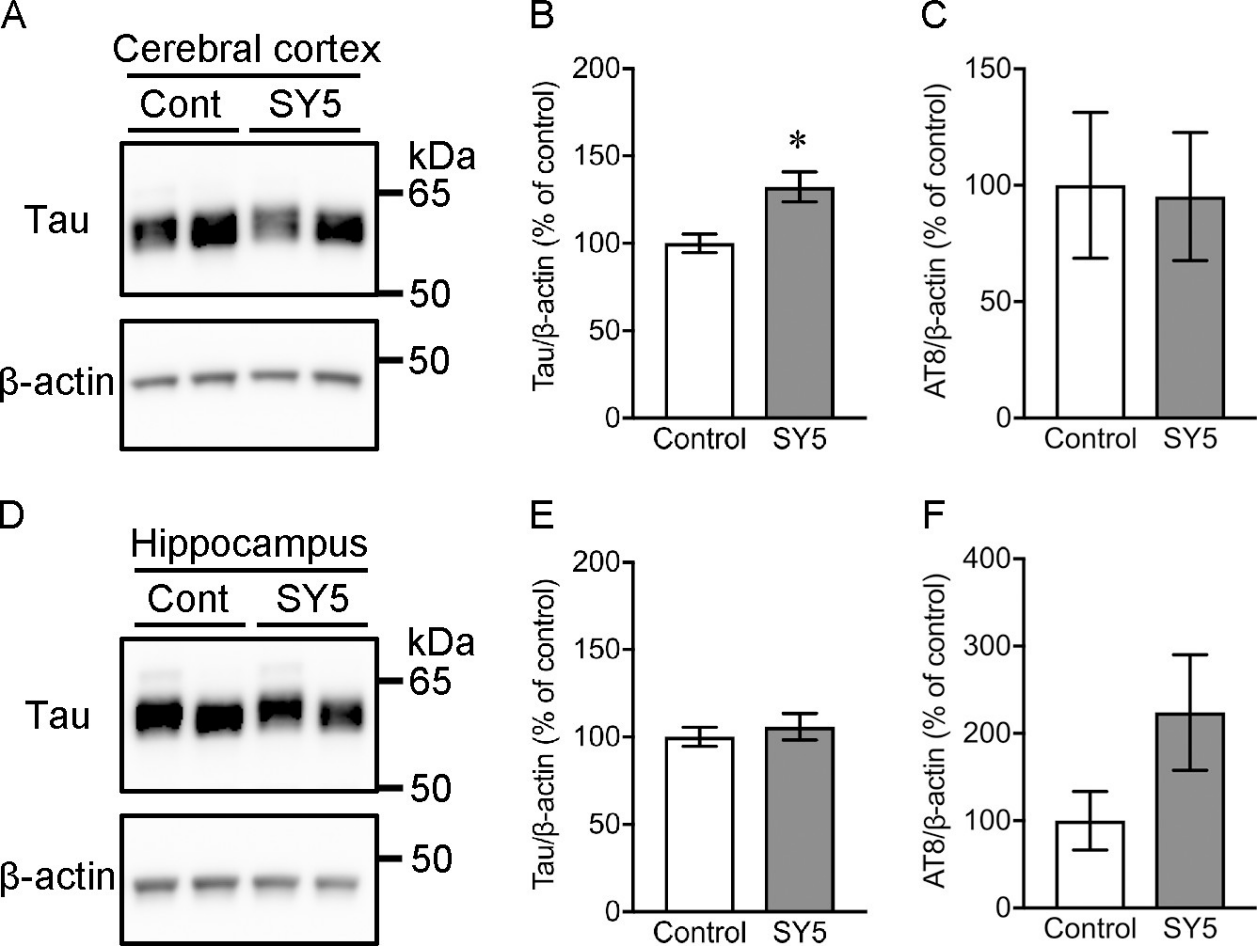
TBS-soluble tau levels in rTg4510 mice. (A, D) Representative western blot images for tau and β-actin in the cerebral cortex (A) and the hippocampus (D). Left and right bands in each group correspond to rTg4510_TxC mice and rTg4510_CxT mice, respectively. (B, C, E, F) Densitometric analysis of the levels of tau and AT8-positive phosphorylated tau in the cerebral cortex (B, C) and the hippocampus (E, F) in rTg4510 mice fed with control chow diet (n = 8) and Shiga-Y5 (SY5)-containing chow diet (n = 7). Data are the mean ± standard error of the mean. Significance (Mann Whitney test): *p < 0.05.

Western blotting detected total tau in TBS-soluble fraction at 55 kDa (Fig 6). The amounts of total tau were significantly higher in the cerebral cortex in rTg4510 mice fed with SY5-containing chow diet compared with rTg4510 mice fed with control chow diet (p < 0.05), although there was no significant difference in the hippocampus between these mice. The levels of AT8-positive phosphorylated tau were further analyzed, however, there were no statistical differences between rTg4510 mice fed with control chow diet and SY5-containing chow diet.

#### Insoluble tau accumulation in rTg4510 mice fed with Shiga-Y5

**Fig 7:**
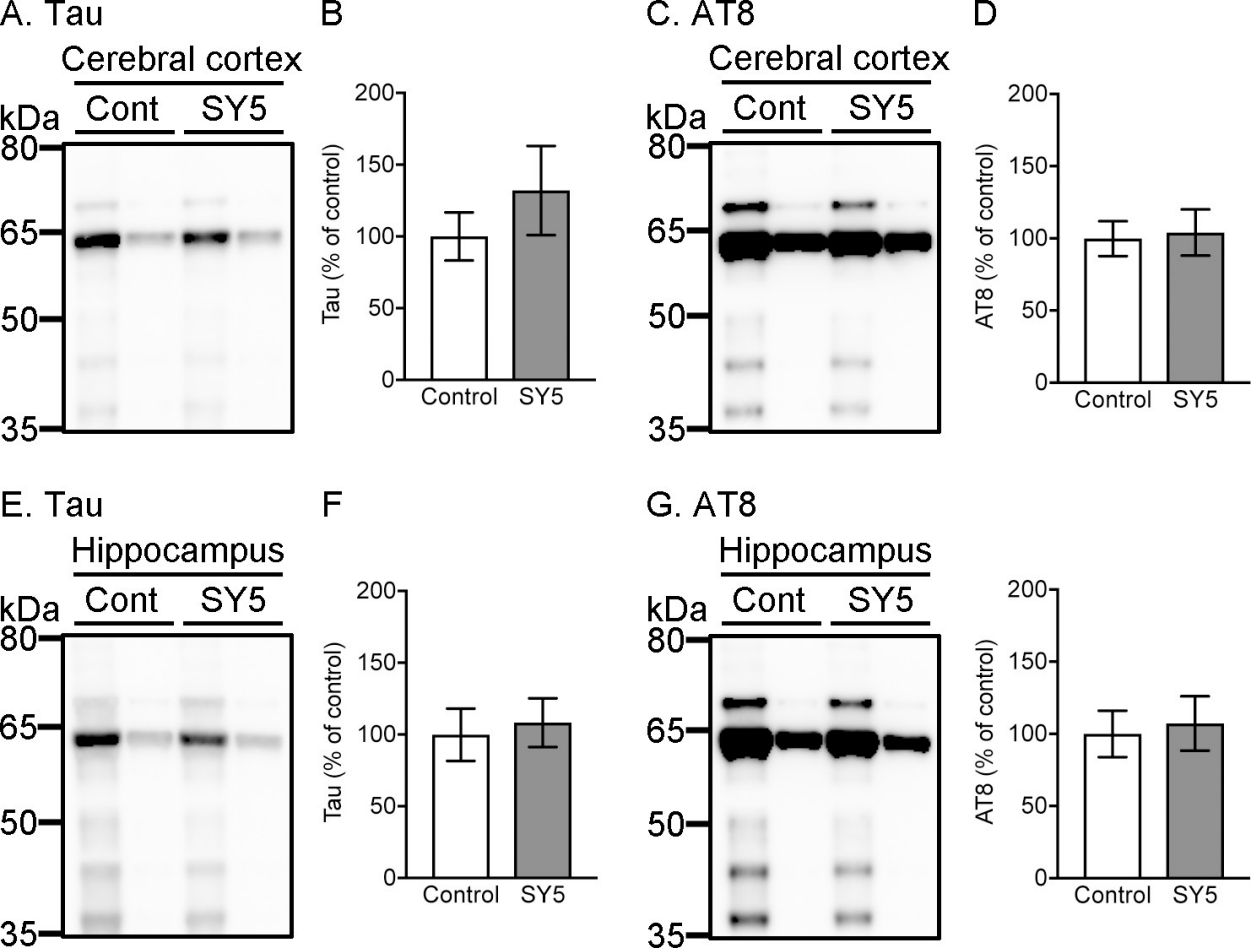
Sarkosyl-insoluble tau accumulation in rTg4510 mice. (A, D) Representative images in western blotting for tau and AT8 in the cerebral cortex (A) and the hippocampus (D). Left and right bands in each group correspond to rTg4510_TxC mice and rTg4510_CxT mice, respectively. (B, C, E, F) Densitometric analysis of tau (B, E) and AT8-positive tau (C, F) levels in the cerebral cortex (B, C) and the hippocampus (E, F) in rTg4510 mice fed with control chow diet (n = 8) and Shiga-Y5 (SY5)-containing chow diet (n = 7). Data are the mean ± standard error of the mean.

Tau and AT8-positive phosphorylated tau in sarkosyl-insoluble fraction were detected at 64 kDa by western blotting (Fig 7). There were no significant differences in the levels of sarkosyl-insoluble tau and AT8 immunoreactive phosphorylated tau in the cerebral cortex and the hippocampus between rTg4510 mice fed with control chow diet and SY5-containing chow diet.

#### Soluble and insoluble tau levels in rTg4510 mice_TxC and rTg4510 mice_CxT

**Fig 8:**
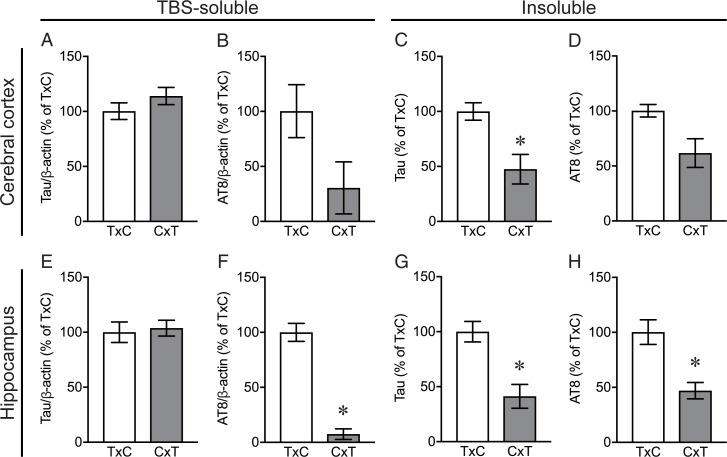
Soluble and insoluble tau levels in rTg4510 mice_TxC and rTg4510 mice_CxT. The levels of tau (A, C, E, G) and AT8-immunopositive phosphorylated tau (B, D, F, H) in the TBS-soluble (A, B, E, F) and sarkosyl-insoluble (C, D, G, H) fractions in the cerebral cortex (A-D) and the hippocampus (E-H) were analyzed in rTg4510_TxC mice fed with control chow diet (n = 4) and rTg4510_CxT mice fed with control chow diet (n = 4). Data are the mean ± standard error of the mean. Significance (Mann Whitney test): *p < 0.05, **p < 0.01.

There were no significant differences in TBS-soluble tau levels in the cerebral cortex and the hippocampus between rTg4510_TxC mice fed with control chow diet and rTg4510_CxT mice fed with control chow diet (Fig 8A and 8E). In contrast, dramatically lower levels of tau in the sarkosyl-insoluble fraction and AT8-positive phosphorylated tau in the TBS-soluble fraction and the sarkosyl-insoluble fraction in the cerebral cortex and the hippocampus were observed in rTg4510_CxT mice fed with control chow diet, compared with rTg4510_TxC mice fed with control chow diet (Fig 8B–8D and 8F–8H).

## Discussion

In the present study, we investigated whether SY5, a curcumin derivative we developed, had therapeutic effects on tau aggregation in rTg4510 mice. However, we could not detect significant differences in behavioral performance and histological and biochemical findings between rTg4510 mice fed with control chow diet and SY5-containing chow diet, except for increasing the level of TBS-soluble tau in the cerebral cortex in rTg4510 mice fed with SY5-containing chow diet. Therapeutic approaches targeting tau aggregation using low molecular-weight chemicals are widely studied, and curcumin and its derivative are promising candidates to inhibit tau aggregation among them [7, 8, 19–21]. Curcumin has been reported to show various beneficial pharmacological properties such as anti-tumor, anti-oxidative, anti-inflammatory effects [22, 23]. Several studies have also reported therapeutic effects of curcumin on Aβ aggregation *in vitro* and amyloid pathology in mouse models of Alzheimer’s disease [24–29]. Furthermore, our previous study revealed that SY5 inhibited Aβ aggregation and improved cognitive deficit in APPswe/PS1dE9 mice [12]. Taken together, it is likely that SY5 is a potential therapeutic agent targeting Aβ aggregation, but not tau aggregation.

It is important to discuss why SY5 did not show the effect on inhibiting tau aggregation while curcumin and other derivatives do. The most major difference in structure between the compounds is the substitution at C-4 position between aromatic groups. SY5 has a methoxycarbonylethyl group at the C-4 position[12], although there is no substitution at the C-4 position of curcumin derivative PE859[7]. One possible reason is therefore that the substitution at C-4 position of SY5 would be a cause of the reduction in the binding activity to tau aggregates. However, further effort is required to elucidate the mechanisms.

Our previous study showed that Shiga-Y5 reaches the brain and binds to amyloid plaques in APP/PS1 mice after intravenous injection [10], indicating blood–brain barrier (BBB) permeability of Shiga-Y5. Conversely, in another study, we could not detect Shiga-Y5 in the plasma and brain of APP/PS1 mice fed with Shiga-Y5 due to the low sensitivity of the method used, although we observed therapeutic effects on amyloid pathology after 6 months’ treatment [12]. Therefore, probably only small amounts of Shiga-Y5 can enter the brain after oral administration and long-term Shiga-Y5 treatment might have a therapeutic effect on tau pathology in rTg4510 mice.

This study investigated the effect of SY5 on protein aggregation and neuronal degeneration in vivo caused by tau protein. Both curcumin and Shiga-Y5 have a 1,7-diphenyl-1,6-heptadiene-3,5-dione structure, the only difference being presence or absence of a side chain at the C-4 position. Shiga-Y5 exists as a keto-enol tautomer due to its 1,3-diketone (or β-diketone) structure. Our previous study showed that similar to curcumin, Shiga-Y5 also inhibits the formation of amyloid-β aggregates curcumin. On the basis of these findings, there is a possibility of Shiga-Y5 inhibiting tau aggregation; therefore, in this study, we investigated this possibility in rTg4510 mice. An in vitro experiment would have helped estimate the effect of Shiga-Y5 on tau aggregation before conducting pharmacological analysis using mouse. However, it was difficult to prepare tau aggregates in vitro. Regarding neuronal degeneration, our results suggested that the SY5 inhibit the key step of cell dysfunction specifically involved in Aβ-induced cell toxicity.

To obtain rTg4510 mice, we used the CaMKII-tTA mouse line on a C57BL/6J background, instead of the original 129S6 background[13, 14]. It has been reported that the original rTg4510 mice displayed impairment of spatial long-term memory in the Morris water maze test, hyperactivity in open-field test, and no impairment of motor function in rotarod test [13, 14, 30–33]. The original rTg4510 mice also showed an increase in total number of arm entries in Y-maze test, while there was no significant difference in spontaneous alteration[31, 33]. In the present study, rTg4510 mice on a partial C57BL/6J strain showed the increase in total number of arm entries in Y-maze test and the impairment in Morris water maze test, as well as no impairments of the performance in rotarod test and the spontaneous alteration in Y-maze test, which corresponded to the findings from the original rTg4510 mice. These findings support those showing no significant alterations in phenotypes between the rTg4510 mice on the partial C57BL/6J strain and the original rTg4510 mice [15].

One of the most interesting findings in the present study was the difference in the phenotypes, such as behavioral dysfunction and tau pathology, between rTg4510 mice on the F1 FVB/NJ x C57BL/6J background (referred to as rTg4510_TxC), which were produced by crossing female tetO-MAPT*P301L mouse line with male CaMKII-tTA mouse line, and on the F1 C57BL/6J x FVB/NJ background (referred to as rTg4510_CxT), which were produced by crossing female CaMKII-tTA mouse line and with tetO-MAPT*P301L mouse line. Although no significant differences were detected in behavioral tests (S2 Fig), tau pathology such as AT8-positive staining and Gallyas silver staining was more severe in rTg4510_TxC than rTg4510_CxT. Biochemical analysis also showed higher accumulation of insoluble tau and AT8-positive phosphorylated tau in rTg4510_TxC, compared with rTg4510_CxT. Since expression levels of tau protein in rTg4510_TxC and rTg4510_CxT, which are generally measured in soluble protein extracts, were almost the same, it is suggested that unknown mechanism to regulate the amount of insoluble tau accumulation is upregulated in rTg4510_TxC mice or downregulated in rTg4510_TxC mice. It is further probable that the difference in the amount of insoluble tau accumulation was attribute to the genes related to sex chromosomes, which are totally different between rTg4510_TxC and rTg4510_CxT. Abnormally hyperhosphorylation is one of the most causative modification in tau to form the insoluble aggregates. There are as many as 85 potential phosphorylation sites (80 Ser or Thr, and 5 Tyr) in the longest tau isoform, and the sites are phosphorylated by proline-directed protein kinases, including cyclin-dependent-like kinase‑5 (CDK5), dual specificity tyrosine-phosphorylation-regulated kinase 1A (DYRK1A), glycogen synthase kinase‑3 (GSK-3β), serine/threonine protein kinases, including calcium/calmodulin-dependent protein kinase II (CAMKII), casein kinase 1 (CK1) family, microtubule affinity regulating kinases (MARKs), mitogen-activated protein kinases (MAPKs,), cyclic AMP-dependent protein kinase (PKA), and non-receptor tyrosine kinases SRC family LCK, SYK, and FYN, the ABL family members ABL1/2[2, 34, 35]. While protein phosphatase 1 (PP1), PP2A, PP2B, PP2C and PP5 have all been implicated in the dephosphorylation of tau, the main regulator of tau phosphorylation is PP2A, which accounts for ~70% of the total tau phosphatase activity in the human brain and its activity is reduced in the AD brain[2, 34, 35]. Among the kinases and phosphatases reported to have major role in tau phosphorylation, we found protein phosphatase 1 regulatory subunit 3F in mouse X chromosome by Mouse Genome Informatics (http://www.informatics.jax.org). Also, there is possibility that other tau modifiers are involved in the insoluble tau accumulation in rTg4510 mice, because tau is modified by truncation, acetylation, N-glycosylation, O-GlcNAcylation, glycation, deamidation, isomerization, nitration, ubiquitination, and methylation[36]. It might be possible to found a novel mechanism to regulate the insoluble tau accumulation in rTg4510 mice in future study.

Tau pathology such as AT8-positive staining and Gallyas silver staining was more severe in rTg4510_TxC than rTg4510_CxT, however, no significant differences were detected in behavioral tests (S2 Fig). It is certainly important to explain the relation between tau pathology and memory impairment in rTg4510 mice. Previous studies showed that abnormal tau phosphorylation and tau pathology in rTg4510 mice are apparent from the age of 2.5 months and that the increase in the extent of pathological changes was age-dependent [14, 37]. Studies also showed memory impairment in rTg4510 mice from the age of 2.5 months and that the deterioration of memory function was age-dependent [13, 14]. Expression of the mutant tau transgene is suppressed in rTg4510 mice using doxycycline through the tet-off system. Santacruz et al [13] also reported that this suppression restored memory function in rTg4510 mice when doxycycline was administered from the age of 5.5 months to 7 or 9.5 months. During this time, although the sarkosyl-soluble tau level decreased, there were no differences in the accumulation of sarkosyl-insoluble tau and the amount of neurofibrillary pathology between doxycycline-treated and doxycycline-untreated rTg4510 mice. Therefore, memory dysfunction in rTg4510 mice is independent of sarkosyl-insoluble tau aggregation. That is, memory recovery in rTg4510 mice implies that reversible neuronal dysfunction, which is possibly caused by sarkosyl-soluble tau oligomers rather than irreversible structural degeneration (e.g., neurofibrillary changes), causes initial memory deficits. In addition, our results showed no direct interaction between the sarkosyl-insoluble tau aggregation level and cognitive impairment in rTg4510 mice. Further studies are required to investigate the differences in the sarkosyl-soluble tau and oligomer levels and cognitive impairment between rTg4510_TxC and rTg4510_CxT mice at an early age in order to understand how the mutant tau transgene induces cognitive impairment.

The importance of strain background in mouse models has been widely observed with regard to expression of disease phenotypes [38, 39], however, it has not previously been reported the effect of swapping female and male parent strain backgrounds on the phenotypes of rTg4510 mice. The present study used male rTg4510 mice at 6 months of age, and therefore we have to carry out further studies using male and female rTg4510 mice with a wider range of age to clarify the effect of swapping female and male parent strain background. This line of investigation could provide novel insights into the mechanisms underlying tau accumulation in tauopathy, which may help to develop therapeutic approaches aiming to modulate tau accumulation in tauopathy.

In conclusion, although curcumin is considered as a promising seed compound targeting tau, SY5 did not improve behavioral dysfunction and tau accumulation in rTg4510 mice. In contrast, we found that swapping female and male parent strain background caused notable difference in the amount of tau accumulation in rTg4510 mice. This finding implies that unidentified mechanism that serves as a key player for tau accumulation exist. Further study would provide important insights into the mechanism underlying tau accumulation in tauopathy.

## Supporting information

S1 Fig Cued navigation test. In Morris water maze test, cued navigation test was conducted on day 8 in wild-type mice fed with control chow diet (n = 8) and Shiga-Y5 (SY5)-containing chow diet (n = 8), and rTg4510 mice fed with control chow diet (n = 8) and SY5-containing chow diet (n = 7). Two wild-type mice fed with control chow diet, one wild-type fed with SY5-containingcho diet and one rTg4510 mouse fed with control chow diet did not reach the platform within 20 seconds.
(EPS)

S2 Fig Comparisons between rTg4510_TxC mice and rTg4510_CxT mice in behavioral tests. Rotarod test (A, B), Y-maze test (C, D), and Morris water maze test (E, F) were conducted in rTg4510_TxC mice fed with control chow diet (n = 4) and Shiga-Y5 (SY5)-containing chow diet (n = 5), rTg4510_CxT mice fed with control chow diet (n = 4) and SY5-containing chow diet (n = 2). Data are the mean ± standard of the mean.
(EPS)
